# Supplementary material for: Escherichia coli Biofilm Formation, Motion and Protein Patterns on Hyaluronic Acid and Polydimethylsiloxane Depend on Surface Stiffness
Source: J Funct Biomater. 2022 Nov 11;13(4):237. doi: 10.3390/jfb13040237 (PMC9680287; doi:10.3390/jfb13040237)

***Escherichia coli* biofilm formation, motion and protein patterns on hyaluronic acid and polydimethylsiloxane depend on surface stiffness**

Annabelle Vigué[1,2], Dominique Vautier[1,2], Amad Kaytoue[1,2], Bernard Senger[1,2], Youri Arntz[1,2], Vincent Ball[1,2], Amine Ben Mlouka[4], Varvara Gribova[1,2], Samar Hajjar-Garreau[5], Julie Hardouin[3,4], Thierry Jouenne[3,4], Philippe Lavalle[1,2], Lydie Ploux[1,2,6]*

[1] INSERM UMR-S 1121 Biomaterial Bioengineering, Centre de Recherche en Biomédecine de Strasbourg, Strasbourg, France

[2] University of Strasbourg, Faculty of Dentistry, Strasbourg, France

[3] Normandie University, UNIROUEN, INSA Rouen, CNRS, Polymers, Biopolymers, Surfaces Laboratory, Rouen, France

[4] PISSARO Proteomic Facility, IRIB, Mont-Saint-Aignan, France

[5] CNRS, Haute Alsace University, Mulhouse Materials Science Institute, Mulhouse, France

[6] CNRS, Strasbourg, France

* Corresponding author: Dr Lydie Ploux, ploux@unistra.fr

Escherichia coli biofilm formation, motion and protein patterns on hyaluronic acid and polydimethylsiloxane depend on surface stiffness

Annabelle Vigué[1,2], Dominique Vautier[1,2], Amad Kaytoue[1,2], Bernard Senger[1,2], Youri Arntz[1,2], Vincent Ball[1,2], Amine Ben Mlouka[4], Varvara Gribova[1,2], Samar Hajjar-Garreau[5], Julie Hardouin[3,4], Thierry Jouenne[3,4], Philippe Lavalle[1,2], Lydie Ploux[1,2]*

[1] INSERM UMR-S 1121 Biomaterial Bioengineering, Centre de Recherche en Biomédecine de Strasbourg, Strasbourg, France

[2] University of Strasbourg, Faculty of Dentistry, Strasbourg, France

[3] Normandie University, UNIROUEN, INSA Rouen, CNRS, Polymers, Biopolymers, Surfaces Laboratory, Rouen, France,

[4] PISSARO Proteomic Facility, IRIB, Mont-Saint-Aignan, France

[5] CNRS, Haute Alsace University, Mulhouse Materials Science Institute, Mulhouse, France

[6] CNRS, Strasbourg, France

* Corresponding author: Dr Lydie Ploux, ploux@unistra.fr

SUPPLEMENTARY MATERIALS

Table S1. Mass measurements of room-hydrated, fully hydrated, and dehydrated HA and PDMS samples.

Materials	w_1 (mg)	w_2 (mg)	w_3 (mg)	Water content (%)	Mean water content ± standard deviation (%)
PDMS-574kPa	55.6	57.3	56.1	2.1	0.9 ± 0.7
	40.6	40.8	40.4	1.0	
	41.5	41.5	41.3	0.5	
	33.3	33.2	33.2	0.0	
PDMS-9kPa	42.9	45.8	41.8	8.7	5 ± 3
	37.5	39.5	39.0	1.3	
	41.8	45.3	42.1	7.1	
	31.1	34.0	31.3	7.9	
	19.7	20.9	20.4	2.4	
HA-2kPa	11.7	75.6	1.6	97.9	97.3 ± 0.2
	24.0	130.8	3.6	97.3	
	20.9	117.4	3.4	97.1	
	36.2	145.9	4.2	97.1	
	34.2	155.0	4.5	97.1	
HA-44Pa	69.0	434.2	2.3	99.4	99.89 ± 0.04

206.7	5,391.2	1.4	100.0
340.0	12,392.8	15.1	99.9
29.2	5,628.3	4.4	99.9
162.4	9,207.8	15.3	99.8

Table S2: Review of published articles on the influence of surface viscoelasticity on bacterial adhesion and biofilm growth on agarose hydrogels (light gray), PDMS materials (dark gray) and other materials (white).

	**Elastic modulus (kPa) (method)**	**Hydration rate or wettability (quantification)**	**Microorganism**	**Culture conditions**	**Influence on adhesion / biofilm formation**	**Reference**
**Gels of alginate, chitosan, modified polyvinyl alcohol (PVA-SbQ) and agarose**	1.6 – 38 (indentation)	Hydrophobic (PVA-SbQ: θe = 92°), moderately hydrophilic (agarose: θe = 49; chitosan: θe = 67) & hydrophilic (alginate: θe = 24°)	*Pseudomonas* sp.	Dewetting step<br>Dynamic<br>Microscopy imaging after rotating biofilm reactor	**No difference** in affinity related to soft/stiffness	Rasmussen el al. 2003[1]
**Polyurethane coating**	1.5 – 1.9 10^6 (AFM; Hertz model)	Hydrophobic (θe = 108-112°) & hydrophilic (θe = 69-75°)	*M. hydrocarbonoclasticus*<br>*Psychrobacter* sp.<br>*H. pacifica*	No dewetting step<br>Dynamic<br>Microscopy imaging in parallel plate (PP) or stagnation point (SP) flow chamber	**Similar** bacterial number on soft and stiff coatings in PP, but fewer on **softer** materials in SP flow chamber (from 8 to 46% less according to species)<br>Less effect on hydrophobic (20%) than on hydrophilic (40%) surface	Bakker et al. 2003[2]
**Polyelectrolyte multilayer films of poly(allylamine) hydrochloride (PAH) and poly(acrylic acid) (PAA)**	800 and 8 10^4 (AFM)	Hydrated (swelling of +14% of the dry thickness)	*E. coli*<br>*S. epidermidis*	Dewetting step<br>Static<br>Plate count method	Fewer bacteria on **softer** materials (at 2h, ~95% less)	Lichter et al. 2008[3]
**Agarose hydrogels & self-assembled polymer vesicles**	11 – 1 000 (nanoindentation)	Hydrated & hydrophilic (not shown)	*E. coli*	No dewetting step<br>Dynamic<br>Microscopy imaging in PP flow chamber	Fewer bacteria on **softer** materials (at 2h, ~75% less)<br>Augmentation of bacterial mobility on softer materials	Cottenye et al. 2012[4]
**Polyelectrolyte multilayer films of HA derivative grafted with vinylbenzyl groups (HAVB) and poly(L-lysine) (PLL)**	30 – 150 (AFM)	Hydrophilic θe = 24±5°	*E. coli*<br>*L. lactis*	Dewetting step<br>Static<br>Microscopy imaging	Fewer bacteria on **softer** films (at 1h, from 17 to 31% less according to species)<br>Higher *E. coli* growth rate on stiffer coatings, but	Saha et al. 2013[5]

					similar *L. lactis* growth rate on stiff and soft coatings	
**Agarose hydrogels**	6.6 and 110 (rheometry)	Hydrophilic (θe = 37-43°)	*Bacillus* sp. *Pseudoalteromonas* sp.	Dewetting step Static Microscopy imaging	Fewer bacteria on **softer** hydrogels (at 3h, 33-66% less according to species)	Guégan et al. 2014[6]
**Poly(ethylene glycol) dimethacrylate (PEGDMA) and agar hydrogels**	44 – 6 489 (indentation)	Hydrophilic (PEGDMA : θe = 61-72° Agar : θe = 15-19°)	*E. coli* *S. aureus*	Dewetting step Static Microscopy imaging	Fewer bacteria (at 2h, 91-95% less according to species) and less biofilm (at 24h, 78% less) on **softer** hydrogels	Kolewe et al. 2015[7]
**PDMS Sylgard 184**	100±20 – 2 600±200 (dynamic mechanical analysis –DMA-)	Hydrophobic (not shown)	*E. coli* *P. aeruginosa*	Dewetting step Static Microscopy imaging	Fewer bacteria on **stiffer** PDMS (at 2h, >95% less)	Song et al. 2014[8]
**Polyacrylamide (PAAm) hydrogels**	0.017±0.005 - 0.654±0.058 (rheometry)	Hydrated (swelling ratio from 29±9 to 82±17; water content >96%)	*S. aureus*	No dewetting step Dynamic Microscopy imaging in PP flow chamber	Fewer bacteria on **stiffer** hydrogels (at 2.5h, >99% less)	Wang et al. 2016[9]
**PDMS Sylgard 184**	100 – 2 600 (DMA)	Hydrophobic (not shown)	*E. coli*	Dewetting step Static Plate count method & Microscopy imaging	Fewer bacteria on **stiffer** PDMS (at 2h, >99% less) More mobility on stiffer PDMS (implication of *motB*)	Song et al. 2017[10]
**PDMS Sylgard 184**	100 – 2 600 (DMA)	Hydrophobic (not shown)	*P. aeruginosa*	Dewetting step Static Plate count method & Microscopy imaging	Fewer bacteria (at 2h, 92% less) and lower levels of c-di-GMP on **stiffer** materials	Song et al. 2018[11]
**Poly(ethylene glycol) (PEG) hydrogels**	1.8 – 1 300 (rheometry)	Hydrated (swelling ratio from 2.20±0.05 to 15.00±0.04)	*E. coli* *S. aureus*	No dewetting step Dynamic Microscopy imaging in PP flow chamber	Lower bacteria residence times on **softer** hydrogels (over 10 min, ~65% less)	Kolewe et al. 2019[12]
**Poly-N-isopropylmethacrylamide (PNiPMAM) based microgel coatings**	21 ± 8 – 346 ± 125 (AFM)	Hydrated (swelling ratio from 1.5 to 2.3)	*S. aureus*	No dewetting step Dynamic Microscopy imaging in PP flow chamber	Fewer bacteria on **softer** microgel (at 4h, 80% less)	Keskin et al. 2019[13]
**PDMS Sylgard 184 with tetrahydrofuran (THF)**	3.4 – 278 10^3 (AFM)	Hydrophobic (θe ~ 120°)	*E. coli* *Pseudomonas* sp.	No dewetting step Static Microscopy imaging	Fewer bacteria on **softer** materials (at 15 min, from 35 to 40% less according to species)	Peng et al. 2019[14]

**PDMS Sylgard 184**	60 ± 1 – 4 520 ± 90<br>(AFM)	Hydrophobic<br>(θe = 110-120°)	*E. coli*<br>*P. aeruginosa*<br>*S. aureus*	Dewetting step<br>Static<br>Microscopy imaging	Fewer *P. aeruginosa* and *E. coli* bacteria on **stiffer** material (at 2h, from 13 to 96% less according to species)	Straub et al. 2019[15]
**PDMS Sylgard 184 & Sylgard 527A&B**	0.26 ± 0.01 and 124 ± 36<br>(rheometry)	Hydrophobic<br>(not shown)	*E. coli*	No dewetting step<br>Static (with & without shaking)<br>Dynamic	Slightly fewer bacteria on **softer** materials without shaking (13% less)<br>**Similar** bacteria number on soft and stiff materials with shaking under low shear stress<br>Bacteria more strongly attached to soft materials	Siddiqui et al. 2019[16]
**PDMS Sylgard 184**	1.3 – 41.8<br><br>(rheology)	Hydrophobic<br>(θe = 110-121°)	*E. coli*	Dewetting step<br>Static<br>Microscopy imaging	**Similar** bacteria number on soft and stiff materials	Qin et al. 2019[17]
**PDMS Sylgard 184**	4 – 103<br>(rheometry)	Non-hydrated<br>(gel fraction = 90-98%)<br>Hydrophobic<br>(θe = 109-119°)	*E. coli*	No dewetting step<br>Dynamic<br>Microscopy imaging in PP flow chamber	Fewer bacteria on **stiffer** material (at 30h and 2h, 23% and 50% less respectively)	Valentin et al. 2019[18]
**PDMS Sylgard 184**	4 – 68 ~~kPa~~<br>(AFM)	Non-hydrated<br>(gel fraction = 75-95%)<br>Hydrophobic<br>(θe = 108-121°)	*E. coli*<br>*P. aeruginosa*<br>*S. epidermidis*	Dewetting step<br>Static<br>Microscopy imaging	Fewer bacteria on **stiffer** materials<br>(at 2h, 50-83% less according to species)	Pan et al. 2020[19]
**PDMS Sylgard 184**	~10 – 2 000<br>(nanoindentation)	Non-hydrated (θe ~ 110-130° ; θe ~ 60° with wrinkle morphology after argon irradiation)	*E. coli*	Dewetting step<br>Static<br>Crystal Violet Staining	**Similar** biomass on soft and stiff materials	[20]

Table S3. Modeling of the Φ functions with $m = 10$ subpopulations for material samples of each type (HA-44Pa, HA-2kPa, PDMS-9kPa and PDMS-574kPa). *D* is diffusion coefficient (μm²/s), r_C is characteristic radius of energy well (μm), *w* is relative weight of subpopulation ($0 \le w \le 1$), <*v*> is mean speed along the trajectories (μm/s), ρ is confinement index. The minor contributions (smallest *w*) are written in small character font.

HA-44Pa

D	rc	w	<v>	ρ
3.162e-3	0.100	0.692	0.039	1.000
6.310e-5	0.126	0.228	8.080e-3	0.365
0.013	0.501	0.063	0.107	0.997
0.251	1.000	0.016	0.369	1.000
0.079	7.943	1.776e-4	0.288	0.134
0.200	79.433	3.931e-5	0.457	3.599e-3
1.585e-5	1584.900	3.930e-5	4.074e-3	7.193e-10
3.981e-3	0.158	1.960e-6	0.052	1.000
0.398	501.190	2.650e-8	0.646	1.807e-4
1.995e-4	1.259	1.540e-9	0.014	0.014

HA-2kPa

D	rc	w	<v>	ρ
1.995e-3	0.158	0.508	0.041	1.000
0.040	0.794	0.227	0.186	0.999
0.100	630.960	0.074	0.324	2.863e-5
3.981e-3	25.119	0.070	0.065	7.190e-4
6.310e-5	0.100	0.067	8.052e-3	0.513
7.943e-3	7.943	0.022	0.091	0.014
3.981e-5	6.310	0.017	6.457e-3	1.140e-4
0.316	794.330	0.016	0.575	5.713e-5
1.995e-3	10.000	1.619e-5	0.046	2.272e-3
0.063	6.310	5.678e-9	0.256	0.165

PDMS-9kPa

D	rc	w	<v>	ρ
3.981e-4	1000.000	0.569	0.018	6.051e-8
0.025	0.200	0.204	0.062	1.000
0.200	0.501	0.136	0.157	1.000
7.943e-3	1.259	0.063	0.078	0.533
0.040	7943.300	0.019	0.177	9.591e-8
0.794	3981.100	0.010	0.790	7.618e-6
3.981e-3	0.126	3.109e-5	0.037	1.000
6.310e-5	0.794	1.192e-5	7.038e-3	0.015
1.585e-4	0.100	6.982e-7	0.011	0.910
0.063	31.623	1.354e-10	0.223	0.010

PDMS-574kPa

D	rc	w	<v>	ρ
1.995e-3	0.100	0.399	0.028	1.000
0.251	0.501	0.181	0.157	1.000
0.079	398.110	0.123	0.250	7.618e-5
6.310e-3	19.953	0.113	0.070	2.406e-3
0.251	10000.000	0.073	0.444	3.818e-7

3.162e-5	1.995	0.067	4.984e-3	1.207e-3
3.981e-4	1000.000	0.044	0.018	6.051e-8
1.000	7943.300	1.524e-4	0.886	2.409e-6
1.585e-5	3.162	1.009e-4	3.528e-3	2.409e-4
0.013	1.259	4.179e-6	0.098	0.701

Figure S1. Photographs of one typical sample of each HA **(A)** and PDMS material **(B)**.

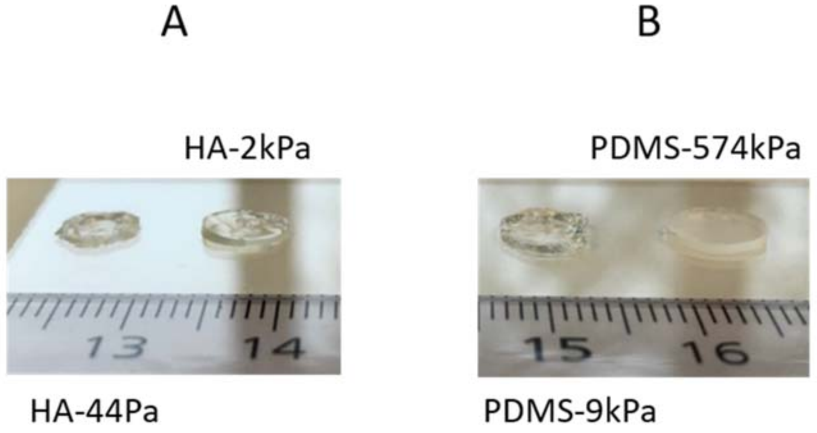

Figure S2. Photographs of a typical 12 mm diameter home-made sample-holder: **(A)** disassembled sample-holder, composed of a lower part on which a 12 mm diameter glass cover plate was placed, an upper part which was screwed on to the lower part, and an O-ring positioned between these two parts to ensure tightness; **(B)** assembled home-made sample-holder. Scale bar 1 cm.

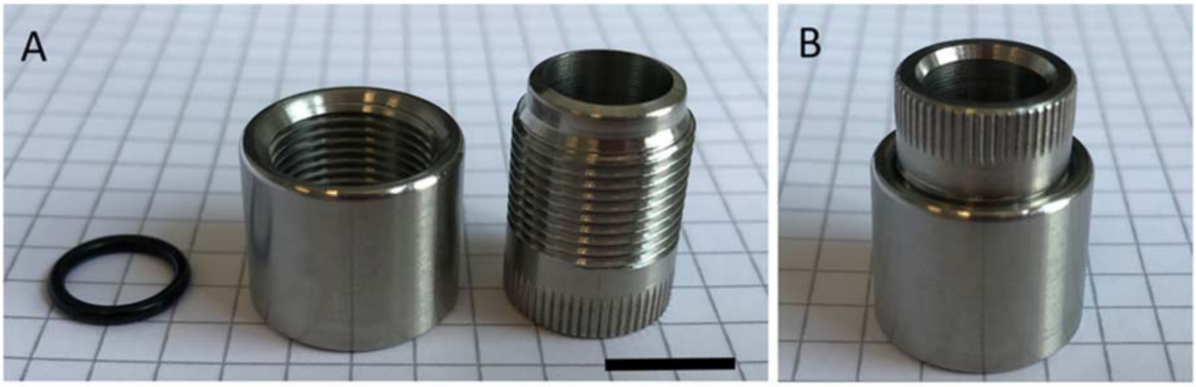

Figure S3. Results of rheometry measurements performed using a rotating KinexusUltra rheometer (Malvern, Great Britain) equipped with a temperature controller set at 25 °C on each type of PDMS and HA material. Measurements were performed in dry conditions in the plate-plate geometry (20 mm parallel plate) for PDMS materials and a plate-cone geometry (176° angle) for HA hydrogels. PDMS disks of 20 mm in diameter and from 1.5 to 2.0 mm in thickness were deposited on the upper plate. The sample was compressed with a maximal deformation of 10% in order to avoid the sample to slip. Measurements were performed in the frequency range between 0.01 Hz and 50 Hz to capture the evolution of the elastic (*G'*) and viscous (*G"*) moduli for 30 min. All measurements were repeated twice. *G'* and *G"* were measured at the highest frequency in the non-glassy phase[1]. Indeed, the linear phase is assumed to correspond to the linear, viscoelastic regime, since the frequency sweep experiments were performed at 1% shear strain. In addition, the materials did not display any damages or ruptures despite compression during the rheometry experiments. Of course, the specification of this behavior would need to conduct rheometry measurements for varying deformation at a constant frequency not essential. However, it is important to be aware that this is here not essential since the objective is the comparative study of the materials at a constant deformation. Results are shown as the average and standard deviation of these measurements. In general, elastic behavior predominated (*G"/G'* from 0.1 to 0.5) except for PDMS-9kPa, which revealed storage and loss modulus of similar values (*G"/G'* ~1). In addition, the elastic modulus of PDMS-574kPa and HA-2kPa was constant in a wide range of frequencies, thus confirming the predominance of elastic behavior. As expected, for both the PDMS and HA materials, the elastic modulus (*G'*) decreased when decreasing cross-linking, ranging from about 150 kPa to less than 1 kPa. PDMS-9kPa and HA-2kPa revealed a similar elastic modulus (6 ± 2 kPa and 2.3 ± 0.8 kPa respectively, measured at the highest frequency in the non-glassy phase[1]). However, their general viscoelastic behaviors were significantly different: HA-2kPa had significant storage

behavior ($G''/G' \sim 0.1$), consistent with the literature[2], while PDMS-9kPa displayed predominantly viscous behavior as already noted by Valentin *et al.*[3].

[1] Techniques for the characterization of physical gels. In Physical Gels from Biological and Synthetic Polymers, Nishinari, K.; Djabourov, M.; Ross-Murphy, S. B., Eds. Cambridge University Press: Cambridge, 2013; pp 18-63, DOI: 10.1017/CBO9781139024136.003.

[2] Ladam, G.; Vonna, L.; Sackmann, E., Micromechanics of Surface-Grafted Hyaluronic Acid Gels. The Journal of Physical Chemistry B 2003, 107 (34), 8965-8971, 10.1021/jp0272872.

[3] Valentin, J. D. P.; Qin, X.-H.; Fessele, C.; Straub, H.; van der Mei, H. C.; Buhmann, M. T.; Maniura-Weber, K.; Ren, Q., Substrate viscosity plays an important role in bacterial adhesion under fluid flow. J. Colloid Interface Sci. 2019, 552, 247-257, 10.1016/j.jcis.2019.05.043.

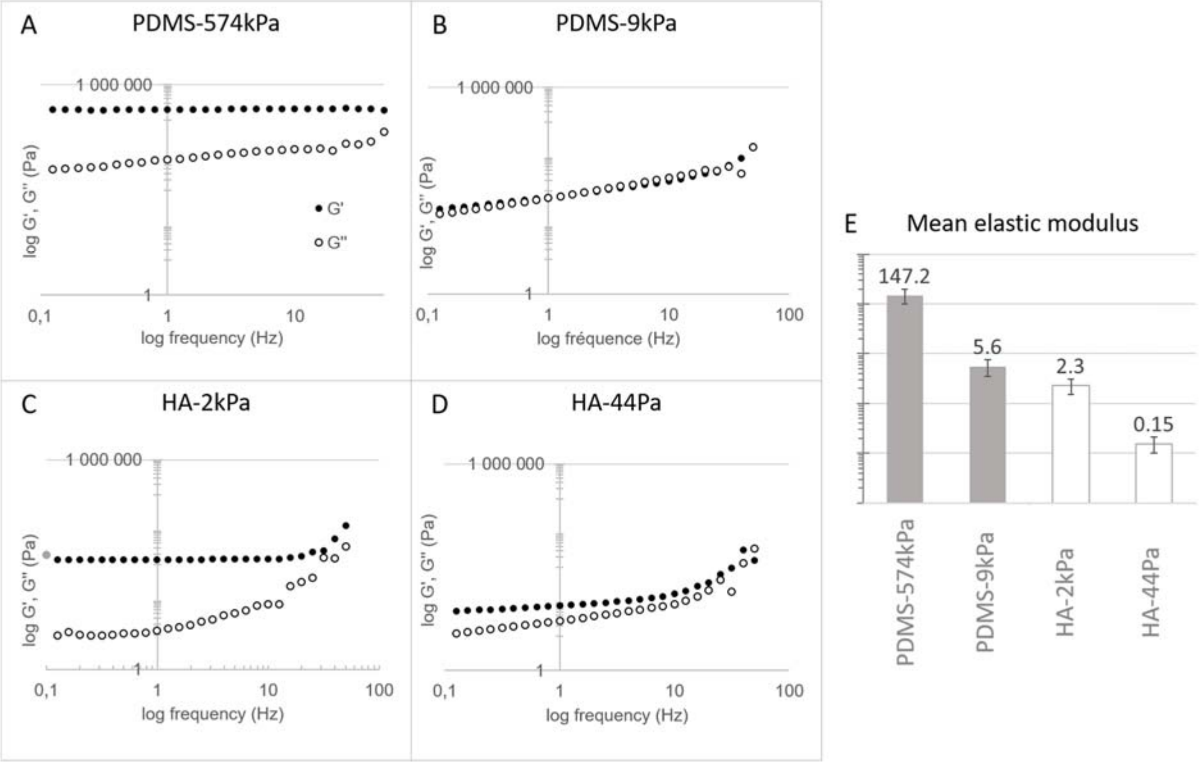

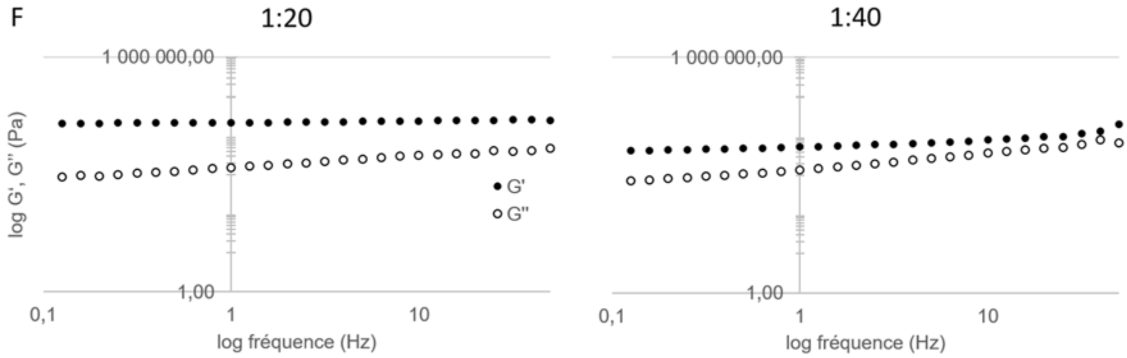
F
1:20
1:40
1 000 000,00
1,00
log G', G'' (Pa)
G'
G''
0,1
1
10
log fréquence (Hz)

Figure S4. Bacterial adhesion on the complete range of PDMS materials with curing agent-to-base mass ratio from 1:80 to 1:5. *: significant difference (p-value< 0.05).

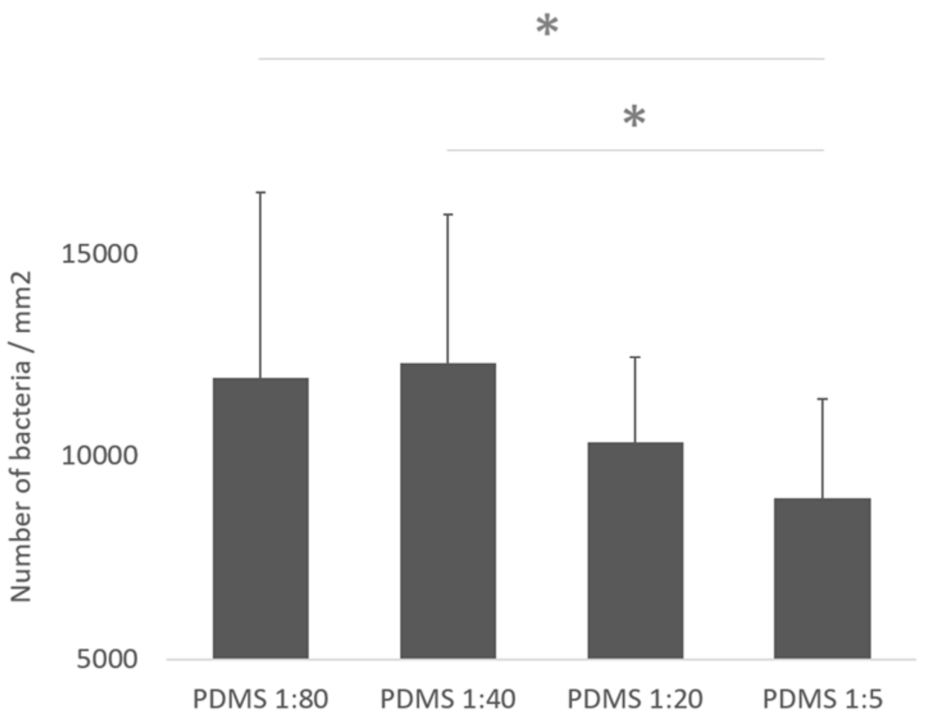

Figure S5. Schematic representation of the dewetting process during washing of the material samples with complete retrieval of the liquid medium. The dewetting front drives out at least "weakly" adhered bacteria (i.e. bacteria 1, 5 and 7 in this representation).

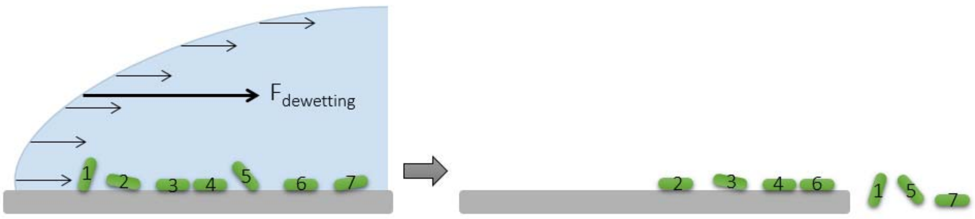

Figure S6. Typical survey **(A)** and high-resolution **(B)** XPS spectra of HA-44Pa, HA-2kPa, PDMS-9kPa and PDMS-574kPa material samples. On PDMS material surfaces, C and O atoms were all involved in C-Si and O-Si bonds respectively as shown by the carbon (C1s) and oxygen (O1s) peaks in high-resolution spectra in accordance with the PDMS chemical structure (**Figure 1**). The C-Si to Si-O ratio (~1.7) was also consistent with the expected ratio of the corresponding bonds in the polymer. In the silicon Si2p peak, a weak signal was attributed to silicon oxides (SiO_2 or $Si(-O)_4$), which probably resulted from the oxidation of a thin layer at the surface of the material. This silicate layer may have a further impact on surface stiffness, which may differ from that of the bulk material [1]. On HA hydrogels, the C-C/C-H component of the C1s peak indicated the presence of the C-C chemical bonds present in the curing agent BDDE (**Figure 1**). The larger amount measured on HA-2kPa compared to HA-44Pa was consistent with the higher quantity of BDDE used, resulting in more cross-linking. In addition, the amount of C-O largely predominates in the C1s peak in relation to the large amount of C-O bonds in both HA and BDDE reagents. The nitrogen atom and acid group of HA expected to be about 400 eV owing to the presence of mesomers of azide, and 289.2 eV, respectively, were not detected in line with their low abundance in the final material. In contrast, a C=O component of low intensity was detected at 288.0 eV, which may contain a low contribution from the O=C-N (288.2 eV) present in the HA molecule. It may also result from ketones formed by the oxidation of the C-OH groups present in the HA and BDDE molecules.

[1] Sim, J. Y.; Taylor, R. E.; Larsen, T.; Pruitt, B. L., Oxidation stiffening of PDMS microposts. Extreme Mechanics Letters 2015, 3, 17-23, https://doi.org/10.1016/j.eml.2015.02.003.

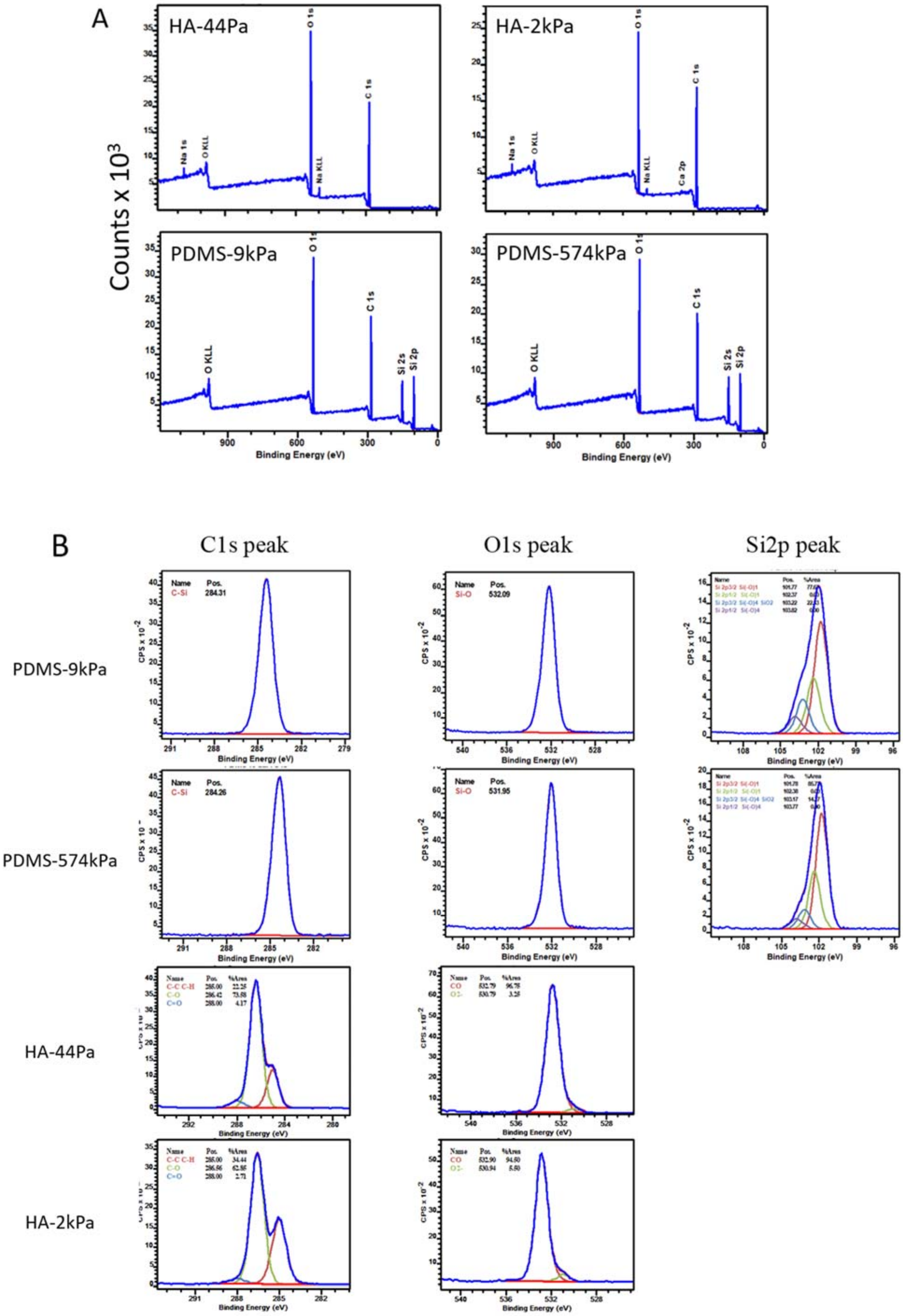
A
HA-44Pa
HA-2kPa
PDMS-9kPa
PDMS-574kPa
Counts x 10^3
O 1s
C 1s
Na 1s
O KLL
Na KLL
Ca 2p
Si 2s
Si 2p
Binding Energy (eV)
B
C1s peak
O1s peak
Si2p peak
PDMS-9kPa
PDMS-574kPa
HA-44Pa
HA-2kPa
Name Pos.
C-Si 284.31
C-Si 284.26
Si-O 532.09
Si-O 531.95
CPS x 10^{-2}
Binding Energy (eV)

Figure S7. SEM Micrograph of *Escherichia coli* SCC1 bacterial cells producing and using pili to adhere to a glass substrate (examples shown by filled arrows) and between each other (examples shown by empty arrows). Some pili between cells were broken, probably due to sample preparation prior to SEM analysis.

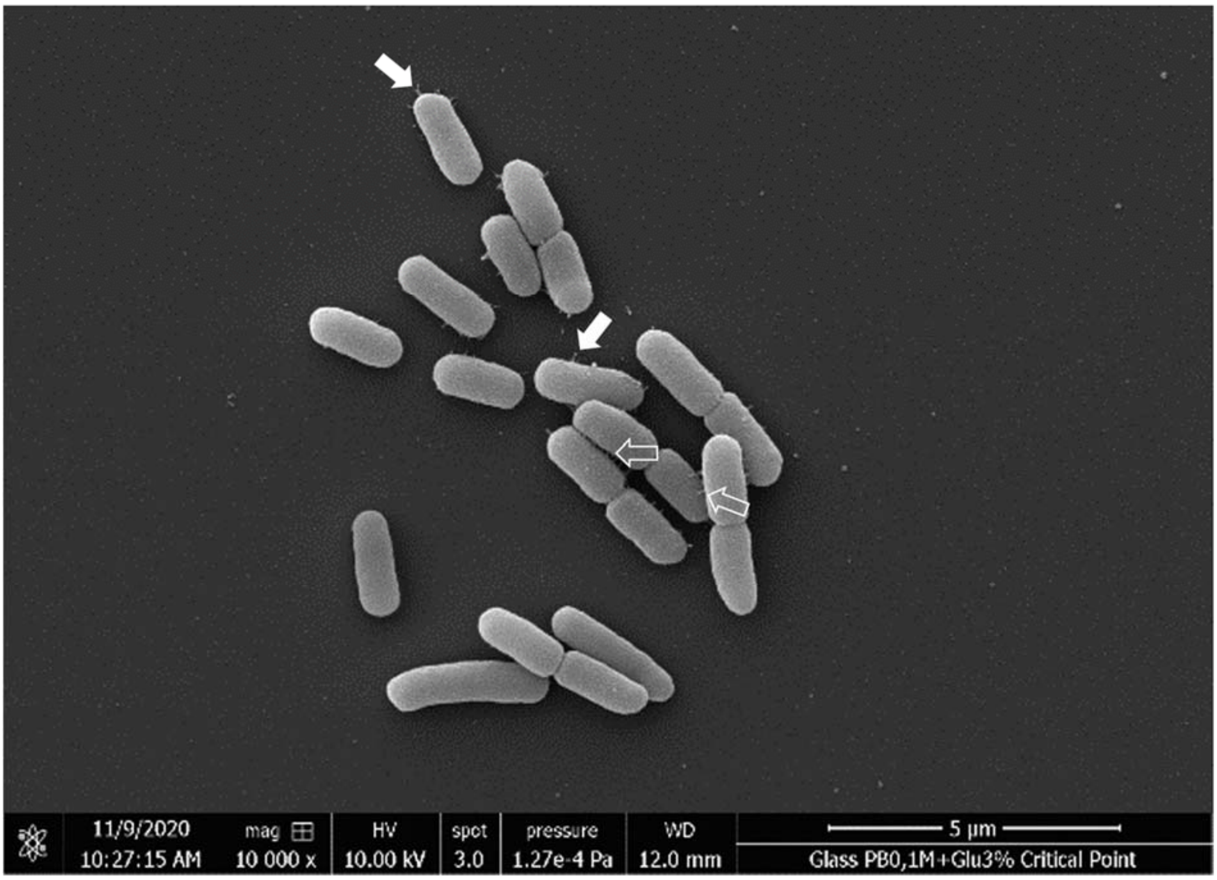

Figure S8. Estimation of the indentation δ produced by a bacterium approximated as a sphere of radius R on a surface characterized by its Young's modulus E using Hertz's law (**Equation S1**). The resulting, calculated values for indentation δ for Young moduli E of 40 Pa, 2 kPa and 500 kPa are 1.8 nm, 0.13 nm and 0.0033 nm, respectively. Bacterium mass ($m_{\text{bacterium}}$ = 1 pg) and volume ($V_{bacterium}$ = 0.6 µm^3) were based on the results reported by Dulbecco and Ginsberg [1], and Prats and Pedro [2]. Indentation δ was calculated as following:

$$\delta = \left[\frac{3F(1-\nu^2)}{4ER^{1/2}}\right]^{2/3} \qquad \textbf{(S1)}$$

where F is the force applied (**Equation S2**) and ν is the Poisson ratio fixed at 0.5 for incompressible materials.

$$F = F_g - F_A \qquad \textbf{(S2)}$$

with F_g, the gravity force applied on a bacterium (**Equation S3**, g, the acceleration of gravity (9.81 m s^{-2})), F_A, the Archimedes' force applied on a bacterium in water (**Equation S4**, with ρ_{water}, the water density (10^3 kg/m^3).

$$F_g = m_{bacterium}\, g \qquad \textbf{(S3)}$$

$$F_A = \rho_{water}\, V_{bacterium}\, g \qquad \textbf{(S4)}$$

[1] Dulbecco, D.; Ginsberg, E., Bacterial Physiology. In Microbiology (Second Edition), Row, H. a., Ed. Maryland, 1973.

[2] Prats, R.; Pedro, M. A. d., Normal growth and division of *Escherichia coli* with a reduced amount of murein. J. Bacteriol. 1989**,** 171(7), 3740-3745, doi:10.1128/jb.171.7.3740-3745.1989.

.

Figure S9: Examples of mean square displacements (MSD) of *E. coli* as a function of time, t, on the diverse materials. In row **A**, left panel, the black symbols correspond to PDMS-574kPa (n = 538) and the red symbols to PDMS-9kPa (n = 542). In row **A**, right panel, the black symbols correspond to PDMS-574kPa (n = 1088) and the red symbols to PDMS-9kPa (n = 975). In row **B**, left panel, the black symbols correspond to HA-44Pa (n = 619) and the red symbols to HA-2kPa (two repeats, n = 1184 and 1622). In row **B**, right panel, the black symbols correspond to HA-44Pa (two repeats, n = 531 and 399) and the red symbols to HA-2kPa (two repeats, n = 161 and 636). Note that the data corresponding to HA-44Pa (left panel) have been multiplied by 10 for the sake of readability. The error bars are standard error of the mean.

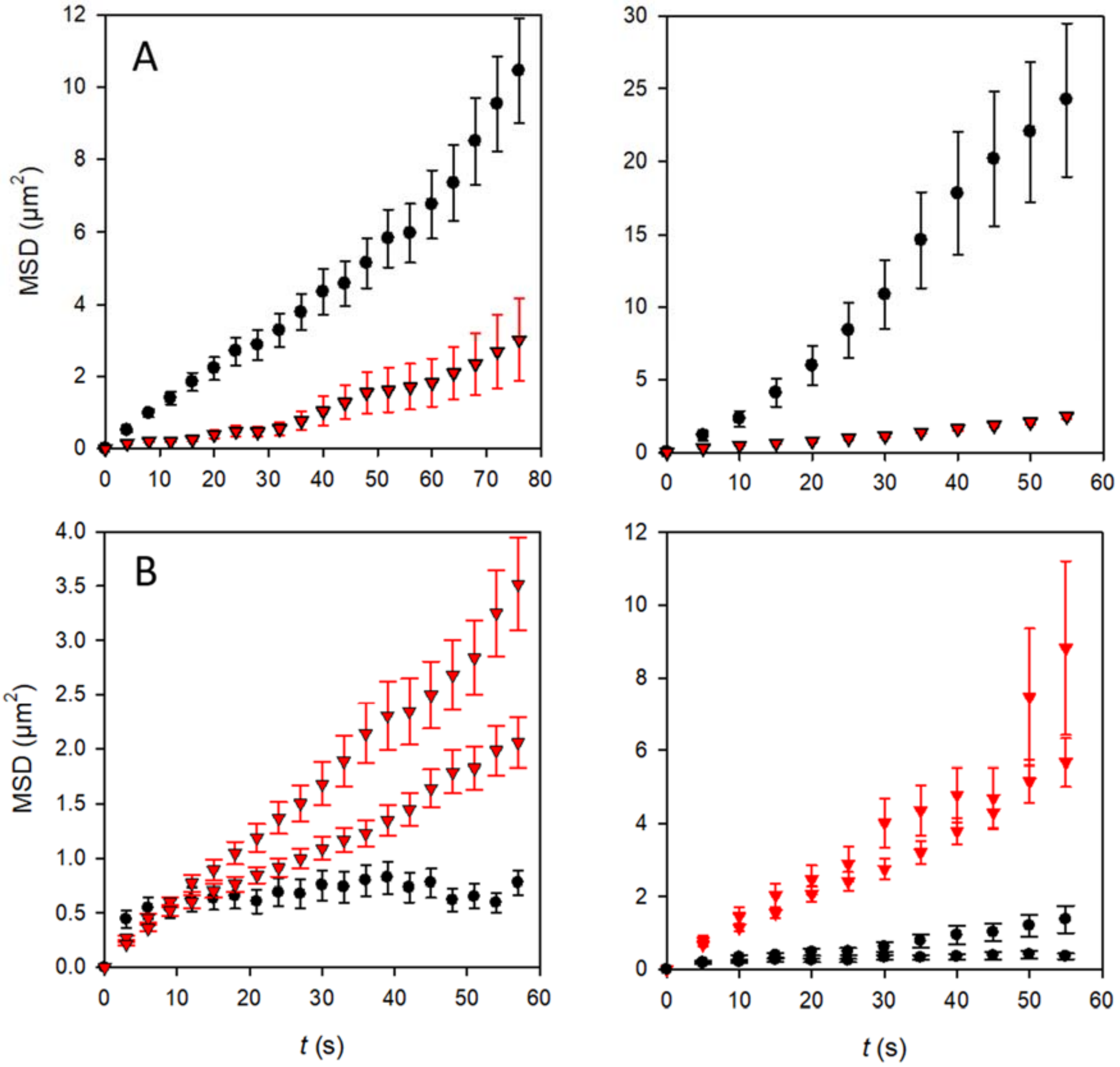

Supplement: Supplementary file 1 [file jfb-13-00237-s001.zip › jfb-1991527-supplementary.pdf]
